# Insulin, glucagon and somatostatin stores in the pancreas of subjects with type-2 diabetes and their lean and obese non-diabetic controls

**DOI:** 10.1038/s41598-017-10296-z

**Published:** 2017-09-08

**Authors:** Jean-Claude Henquin, Majeed M. Ibrahim, Jacques Rahier

**Affiliations:** 1 Unit of Endocrinology and Metabolism, Faculty of Medicine, University of Louvain, Brussels, Belgium; 2 Department of Pathology, Faculty of Medicine, University of Louvain, Brussels, Belgium

## Abstract

In type-2 diabetes, both insufficient insulin and excessive glucagon secretion contribute to hyperglycemia. We compared insulin, glucagon and somatostatin stores in pancreas obtained at autopsy of 20 lean and 19 obese non-diabetic (ND), and 18 type-2 diabetic (T2D) subjects. From concentrations and pancreas weight, total content of hormones was calculated. Insulin content was 35% lower in T2D than ND subjects (7.4 versus 11.3 mg), whereas glucagon content was similar (0.76 versus 0.81 mg). The higher ratio of glucagon/insulin contents in T2D was thus explained by the decrease in insulin. With increasing BMI of ND subjects, insulin and glucagon contents respectively tended to increase and decrease, resulting in a lower glucagon/insulin ratio in obesity. With aging, insulin and glucagon contents did not significantly change in ND subjects but declined in T2D subjects, without association with the duration of diabetes or type of treatment. The somatostatin content was lower in T2D than ND subjects (0.027 versus 0.038 mg), but ratios somatostatin/insulin and somatostatin/glucagon were not different. In conclusion, insulin stores are about 1/3 lower in T2D than ND subjects, whereas glucagon stores are unchanged. Abnormal secretion of each hormone in type-2 diabetes cannot be attributed to major alterations in their pancreatic reserves.

## Introduction

Glucose homeostasis mainly relies on the opposite hypoglycemic and hyperglycemic properties of insulin and glucagon. In type-2 diabetic (T2D) subjects, hyperglycemia is largely the consequence of insufficient insulin secretion and excessive glucagon secretion in a context of insulin resistance^1–3^. These secretory abnormalities may result from isolated or combined alterations in the function and number of pancreatic β- and α-cells. Three pioneer groups used bioassays to compare insulin concentrations in extracts of the pancreas from non-diabetic (ND) and diabetic subjects^4–6^. Subsequent studies used radioimmunoassays to measure insulin and/or glucagon in the human pancreas^7–17^. However, those combining pancreatic insulin and glucagon measurements in ND subjects^11–14^, or comparing pancreatic insulin in ND and T2D subjects^8, 9, 16, 17^ remain infrequent. Pancreatic glucagon and somatostatin stores have not previously been measured in T2D subjects.

In this paper, we report measurements of insulin, glucagon and somatostatin in the pancreas of 18 T2D subjects and 39 ND subjects. The number of the latter also permitted assessment of the impact of obesity by comparing 20 lean non-diabetic subjects (LND) and 19 obese non-diabetic subjects (OND).

## Results

### Hormone concentrations in the pancreas

**Figure 1:**
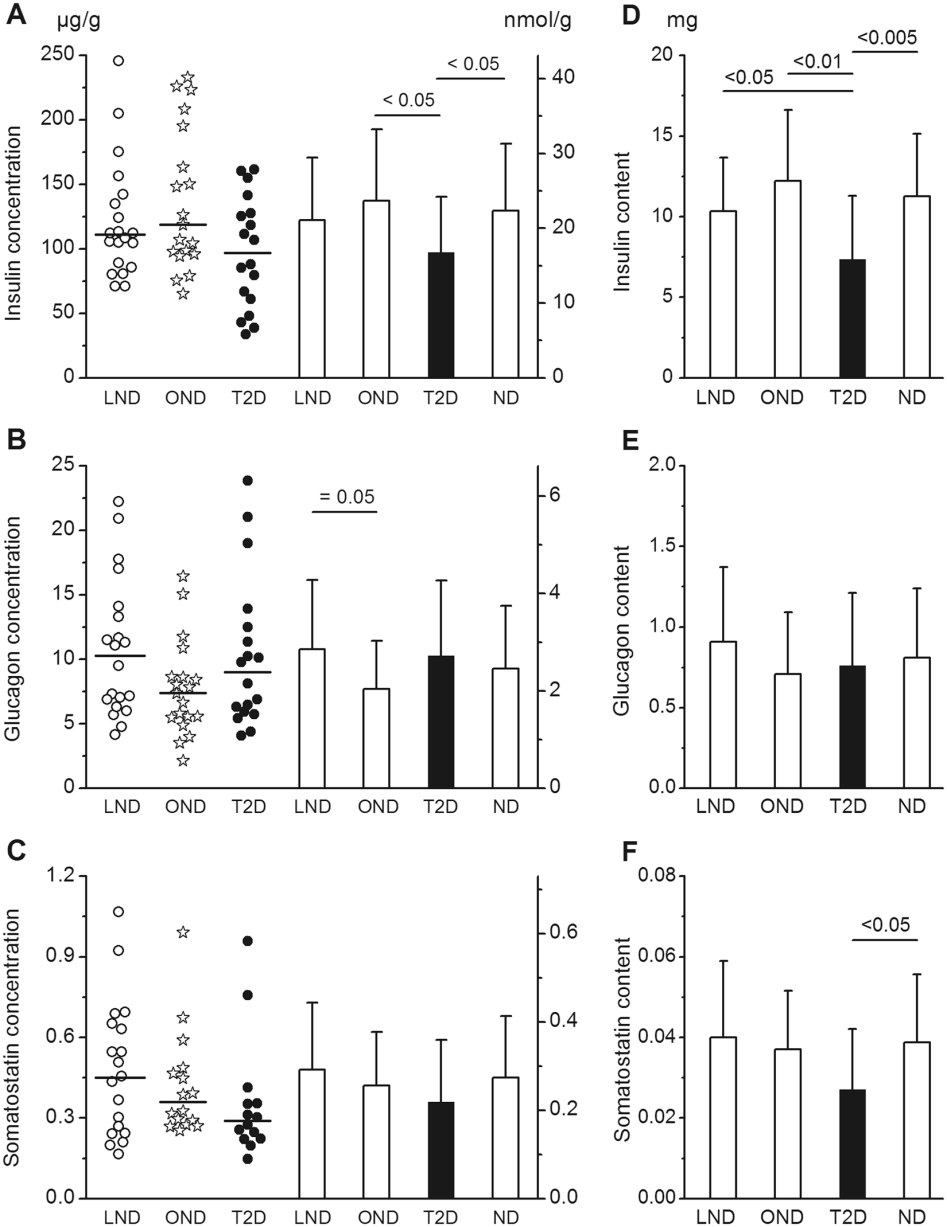
Pancreatic insulin, glucagon and somatostatin concentrations and contents. Panels (A–C) show hormone concentrations (in µg/g or nmol/g) in the body of the pancreas of 20 lean non-diabetic (LND), 19 obese non-diabetic (OND) and 18 type-2 diabetic (T2D) subjects. Individual values are shown as scatter plots with medians. Columns show mean values ± SD. Right-hand columns show mean results for all (lean and obese) ND subjects. Panels (D–F) show mean insulin, glucagon and somatostatin contents (in mg) of the pancreas. In each subject hormone content was obtained by multiplying the concentration measured in the body by the weight of the whole pancreas. Significant differences between groups shown above columns were calculated by Anova and confirmed by non-parametric tests, except for insulin concentration in T2D versus ND subjects (*P* = 0.065).

Pancreatic concentrations of insulin, glucagon and somatostatin were characterized by a great inter-subject variability (3.8- to 7.6-fold) in the three groups of subjects (Fig. 1A–C). Pancreatic insulin concentrations were not significantly different in LND and OND subjects (122 ± 48 and 138 ± 55 µg/g) (Fig. 1A). In T2D subjects, the mean concentration of insulin (97 ± 43 µg/g) was slightly lower than in the groups of OND or of all ND subjects. Notably, however, two thirds of T2D subjects had a pancreatic insulin concentration within the range of concentrations in ND subjects (Fig. 1A).

The mean pancreatic glucagon concentration in T2D subjects (10.3 ± 5.8 µg/g) was not different from that in ND subjects (9.3 ± 4.8 µg/g) or in the two subgroups of controls (Fig. 1B). It was marginally lower in OND than LND subjects. The mean somatostatin concentration in T2D subjects (0.37 ± 0.25 µg/g) was not significantly different from that in ND subjects (0.46 ± 0.24 µg/g) or in the two subgroups of controls (Fig. 1C).

### Hormone contents of the pancreas

In the whole group of ND subjects, the weight of the pancreas averaged 90.8 ± 22.6 g, and did not correlate with age (r = −0.301, *P* = 0.064) or BMI (r = 0.209, *P* = 0.210), but increased slightly with body weight (r = 0.366, *P* = 0.026). It was 16% lower (76.2 ± 20.0 g, *P* < 0.05) in T2D subjects. From the weight of each pancreas and measured hormone concentrations, the total content of each hormone could be calculated (Fig. 1D–F). In the group of 39 ND subjects the insulin content averaged 11.3 ± 3.9 mg and it was 35% lower in T2D subjects (7.4 ± 3.9 mg, *P* < 0.005). The decrease remained significant when compared with LND and OND subjects separately. Among controls, the insulin content was not significantly different between OND and LND subjects (Fig. 1D).

The pancreatic glucagon content averaged 0.81 ± 0.43 mg in ND subjects and 0.76 ± 0.45 mg in T2D subjects. There was no difference between glucagon contents in T2D subjects and any of the other groups (Fig. 1E). In ND subjects, the pancreatic somatostatin content averaged 0.038 ± 0.017 mg. It was 29% lower in T2D subjects (0.027 ± 0.015 mg, *P* < 0.05), but the difference was not significant when compared with the subgroups of LND and OND subjects (Fig. 1F).

### Pancreatic hormone contents versus age

**Figure 2:**
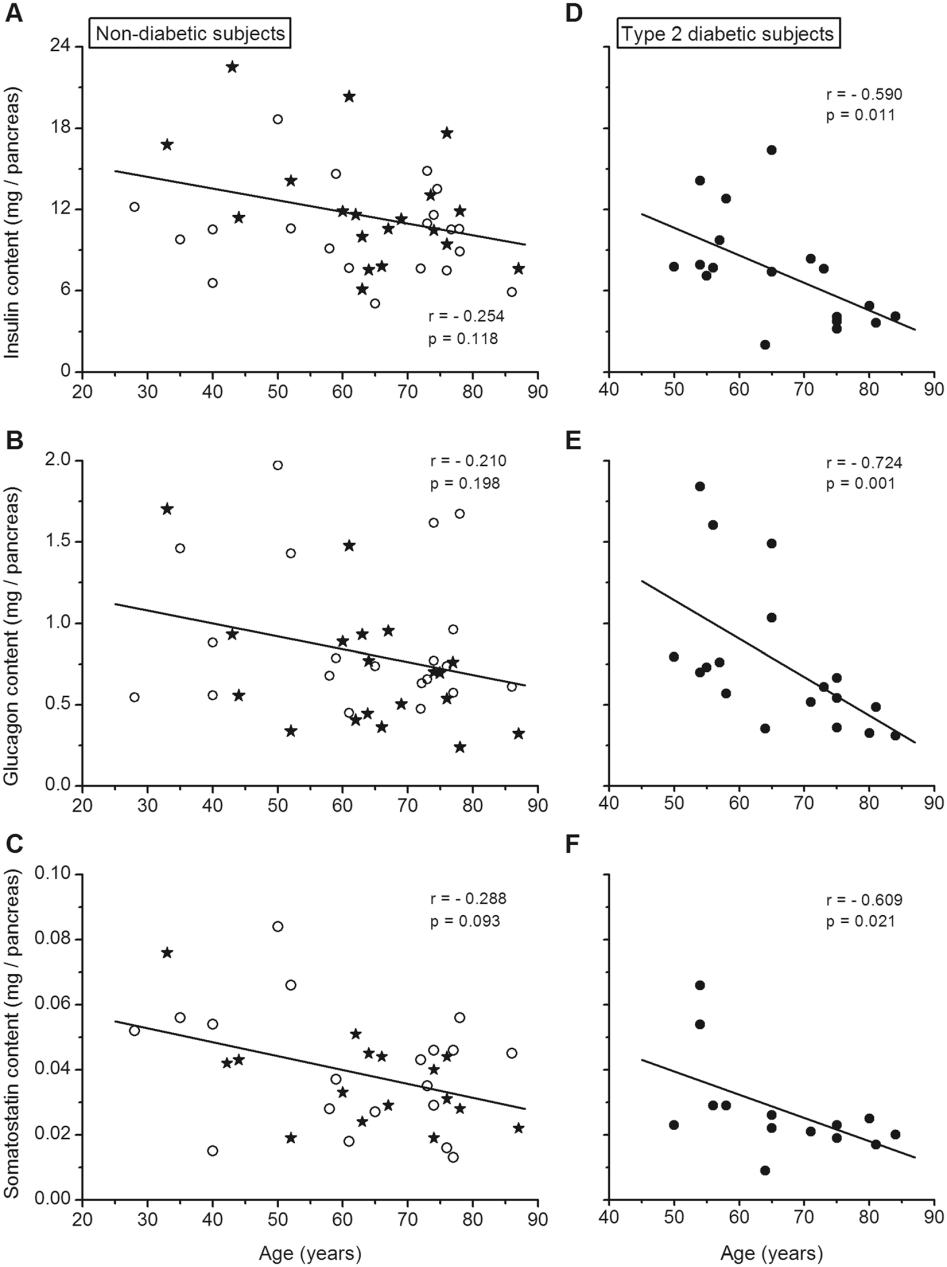
Correlations between the insulin, glucagon and somatostatin content of the pancreas and the age of the subjects. Panels (A–C) show results for lean (open circles) and obese (filled stars) non-diabetic subjects by different symbols. Panels (D–F) show results for type-2 diabetic subjects. Correlation coefficients were calculated by the test of Spearman.

In the whole group of ND subjects, insulin, glucagon and somatostatin contents of the pancreas tended to decrease slightly with age, but the correlations did not reach statistical significance (Fig. 2A–C). There was also no significant correlation when the subgroups of LND and OND were tested separately. In addition, mean insulin, glucagon and somatostatin contents were not statistically different in ND subjects below and above 65 years. In contrast, aging was associated with a decrease in pancreatic content of the three hormones in T2D subjects (Fig. 2D–F). This was confirmed by a significant difference in mean insulin and glucagon contents in T2D subjects below and above 65 years (*P* ≤ 0.01).

### Pancreatic hormone contents versus BMI

**Figure 3:**
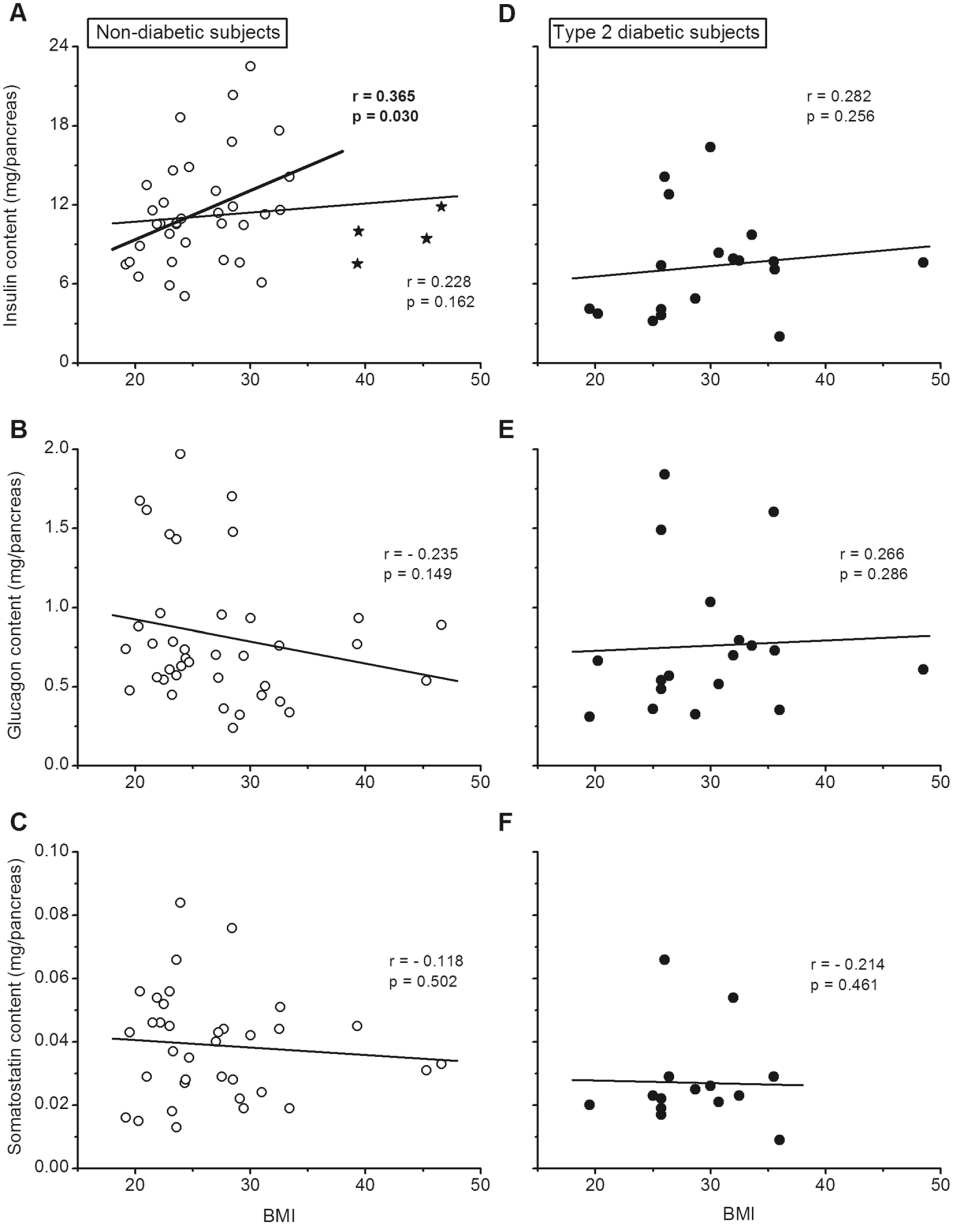
Correlations between the insulin, glucagon and somatostatin content of the pancreas and the BMI of the subjects. Panels (A–C) show results for non-diabetic subjects. (**A**) The correlation calculated for all subjects is shown by the thin line. The correlation calculated after exclusion of the 4 subjects with a BMI above 38 (stars) is shown by the thick line. Panels (D–F) show results for type-2 diabetic subjects. Correlation coefficients were calculated by the test of Spearman.

In ND subjects, no correlation was found between the insulin, glucagon or somatostatin content and BMI (Fig. 3A–C) or body weight. There was, however, a significant positive correlation between the insulin content and BMI when the 4 most obese subjects (BMI > 38) were excluded from the analysis (Fig. 3A). In T2D subjects, insulin, glucagon and somatostatin contents were also unrelated to BMI (Fig. 3D–F) or body weight.

### Duration of diabetes, type of treatment and pancreatic hormone contents

Clinical duration of diabetes (time since diagnosis) was known in 14/18 T2D subjects (Supplementary Table 1). It did not correlate with pancreatic insulin content (r = −0.386, *P* = 0.172), glucagon content (r = −0.294, *P = *0.308) or somatostatin content (r = −0.589, *P* = 0.081). Of the 18 T2D subjects, 8 received insulin alone or with an oral drug, and 8 received a sulfonylurea alone or with insulin (Supplementary Table 1). Treatment with insulin or sulfonylurea did not affect insulin, glucagon or somatostatin concentration or content of the pancreas (Supplementary Fig. 1).

### Ratios of hormone concentrations in the pancreas

**Figure 4:**
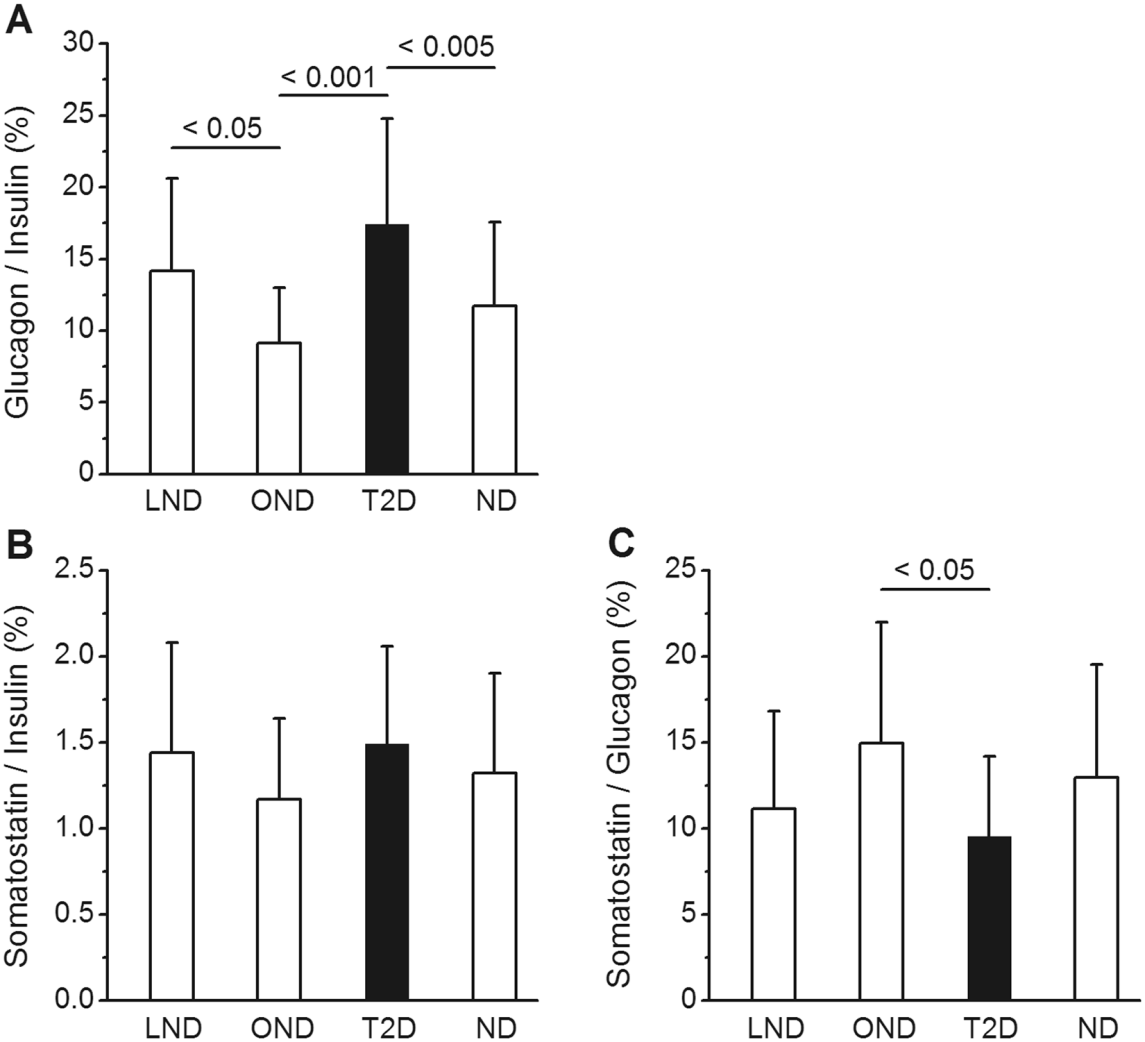
Molar ratios of hormone concentrations in the pancreas. Results are shown for lean non-diabetic subjects (LND), obese non-diabetic subjects (OND), all non-diabetic subjects (ND) and type-2 diabetic subjects (T2D). These ratios were calculated from hormone concentrations in nmol/g shown in Fig. 1 and are expressed as percentages. Values are means ± SD. Significant differences between groups shown above columns were calculated by Anova and confirmed by non-parametric tests.

These ratios were calculated using molar concentrations of each hormone (right-hand scales in Fig. 1A–C) and were expressed as percentages. The same results would be obtained from calculations based on hormone content. In the group of 39 ND subjects the glucagon/insulin ratio averaged 11.7 ± 5.8%. It was higher (17.4 ± 7.7%) in T2D subjects (Fig. 4A). Among controls, the ratio was lower in OND than LND subjects (Fig. 4A) because glucagon and insulin concentrations in OND tended to be lower and higher, respectively (Fig. 1A and B). In ND subjects the somatostatin/insulin ratio averaged 1.32 ± 0.58% and it was similar in the two subgroups of LND and OND subjects and in T2D subjects (Fig. 4B). The somatostatin/glucagon ratio averaged 12.9 ± 6.6% in ND subjects. It was slightly lower in T2D than OND subjects (Fig. 4C). None of these ratios was significantly correlated with age or BMI.

## Discussion

The present study, based on data obtained more than 25 years ago, suffers from one limitation that we were unable to rectify. The still accessible clinical records (autopsy notes) did not include primary results of blood glucose or HbA1c assays formally excluding diabetes in all ND subjects. Another potential problem is the heterogeneity of islet distribution, with a higher islet volume density in the tail than in the head and body of the pancreas^17, 18^. In two subsets of the studied subjects we also obtained samples from the head or the tail, and found that insulin concentrations were ~36% lower in the head than in the body and ~30% lower in the body than in the tail, in agreement with previous studies^5, 12, 14^. Glucagon concentrations were also higher in the body than the head but were not compared between body and tail. One consequence of these regional variations is that calculations of hormone content of the pancreas, based on the intermediate values measured in the body region, only provide reasonable estimates. Optimally, samples from the three regions of the pancreas should be extracted in parallel.

Comparing biochemical measurements of pancreatic hormones with quantitative morphological studies of the endocrine pancreas is not straightforward. The major pitfall is that immunohistochemical techniques detect the presence but do not usually measure the concentration of hormones in cells. Yet, relative changes in the volume density of a given cell type can be compared with relative changes in the concentration of the corresponding hormone. Theoretically, comparisons with the hormone content of isolated islets should also be instructive. However, the pitfall is that the cell composition varies with islet size^19–21^. Selected isolated islets may not always be representative of the whole islet population.

Pancreatic insulin and glucagon concentrations displayed a marked variability in ND subjects, in keeping with the known inter-subject variations in islet volume density^17^. In the whole group of 39 ND subjects, mean pancreatic insulin concentration (130 µg/g) was slightly above the average of previously published values (~100 µg/g, range 53–199)^4–6, 8–14, 16, 17^. The glucagon concentration (9.3 µg/g) was within the range of reported values (7–18 µg/g)^7, 11–15^. From these measurements we calculated that the pancreas of ND subjects contains about 11.3 mg insulin (~325 IU) and 0.81 mg glucagon. In seven immunohistochemical studies of the pancreas from ND subjects, the ratio of α-cell/β-cell volume densities averaged 31% (range 19–44)^18, 20, 22–26^. This ratio is higher than the molar ratio of glucagon to insulin contents which averaged 11.7% (7.6% in µg/g). The discrepancy is even greater with isolated islets in which a glucagon/insulin ratio of only 3% has been measured^27^. The obvious implication is that α-cells contain less glucagon than β-cells contain insulin. That is indeed the case^28^, but quantitative comparisons would be hazardous because α-cells are smaller than β-cells and the proportion of α/β cells increases with islet size^20–22^. Further studies are necessary to resolve the issue.

Aging is associated with deterioration in glucose tolerance^29^. Both β- and α-cell masses have been found to decrease slightly with aging in ND subjects^17, 20, 30^ although one study measured no change in the β-cell mass^31^. In partial contrast with these morphological observations, pancreatic insulin and glucagon contents did not significantly decline with age and mean contents were not significantly different between ND subjects below or above 65 years of age. In a study based on bioassays, a small non-significant decline of pancreatic insulin stores was measured in ND subjects between the second and eighth decades^5^. No correlation was found between the insulin content of isolated islets and age between 19 and 64 years^32^, and a slight increase (20%) was measured in islets isolated from donors >60 years compared with donors <50 years^33^. Overall these observations indicate that the decrease in insulin secretion occurring in older subjects^34^ is not attributable to insufficient insulin reserves.

Whereas isolated obesity notoriously increases basal and stimulated insulin secretion^35, 36^, its influence on glucagon secretion is uncertain^37, 38^. The α-cell mass does not change with BMI^20, 39^. Compared with LND subjects, the β-cell mass is ~50% larger in very obese subjects (mean BMI of 35)^31^ but is much less increased (~20%) or even unchanged in cohorts with milder obesity (mean BMI of 29.9 and 28.5)^17, 39^. Islets isolated from lean and obese donors contain similar amounts of insulin^32, 33^. The impact of obesity on pancreatic insulin stores has not been examined in previous studies. We found that pancreatic insulin concentration and content were similar in OND and LND subjects (BMI 32.5 vs 22.4). The slight difference in insulin content (19%) was of the same magnitude as the increase in β-cell mass in OND, but failed to reach significance perhaps because the sample size was too small. Intriguingly, however, a positive correlation between pancreatic insulin content and BMI was disclosed when the 4/19 OND subjects with the highest BMI (>38) were excluded from the analysis. It is possible that insulin synthesis fails to compensate adequately for the high secretory demands in extreme obesity. In contrast to insulin, pancreatic glucagon concentrations were slightly lower in OND than LND subjects, which resulted in a lower glucagon/insulin ratio in the pancreas of OND subjects. Overall, insulin and glucagon stores, and β-cell and α-cell masses change relatively little with increasing BMI in ND subjects.

Insufficient insulin secretion is a key factor in the pathogenesis of type-2 diabetes^1, 40^, but the underlying causes remain unclear. The β-cell volume density is lower in T2D than ND subjects^17, 18, 23–26, 41, 42^, but the magnitude of the reported decrease varies between 24 and 60%, and the overlap between ND and T2D values is important^17^. In the present cohort of T2D subjects, mean pancreatic insulin concentration was 25% lower than in ND subjects, also with an important overlap between the two groups. Total insulin content was 35% lower owing to the slightly smaller size of the pancreas in T2D subjects. These differences are similar to those found in our previous report^17^ but smaller than the average 54% decrease in others series^4–6, 8, 16^. Notably, the greatest differences were found in the oldest studies, in which the characteristics of diabetic subjects were uncertain. For example, in the large cohort of Wrenshall^5^, the pancreatic insulin concentration was much lower in diabetic subjects aged 20–40 years than in those above 55 years. Several groups have isolated pancreatic islets from diabetic organ donors, but only few independent studies compared the insulin content of islets from significant numbers of T2D and ND donors. The decrease measured in T2D islets averaged 26% (14–35%)^27, 43, 44^. In our group of T2D subjects, pancreatic insulin stores did not correlate with BMI but decreased with age, which suggests that a progressive lowering of insulin reserves might contribute to age-dependent aggravation of type-2 diabetes^29^. We did not find links between insulin stores and duration of clinical diabetes (true duration of the disease cannot be determined) or type of treatment. Studies of larger cohorts would however be useful to ascertain these findings.

Because our insulin assay cross-reacted with proinsulin, reported concentrations of insulin are overestimated. However, the error is likely to be small. Previous studies have shown that the proinsulin/insulin ratio is only 2–3% in human normal pancreas^11^ and purified β-cells^45^. Whether this proportion is modified in type-2 diabetes and obesity is not known. We are aware of only one report succinctly stating that there is no significant difference in proinsulin proportion between pancreatic extracts from diabetic and non-diabetic subjects^9^.

The mechanisms causing excessive glucagon secretion in type-2 diabetes remain unclear^2, 3, 46, 47^. Owing to the decrease in β-cell volume density, an increase in the relative proportion of α-cells/β-cells has consistently been observed in the pancreas of T2D subjects. However, the absolute α-cell mass was variably found to be decreased^21^, increased^25^ or unchanged^20, 26^. Our unprecedented measurements of pancreatic glucagon in T2D subjects showed a high inter-subject variability, no relation to BMI and a decrease with aging, similar to that of insulin. Most importantly, pancreatic glucagon content was similar in T2D and ND subjects, which indicates that the higher glucagon/insulin ratio was caused by the decrease in insulin content. In one study of islets isolated from 6 T2D donors, the glucagon content was three-fold higher than in islets from ND donors^27^. The discrepancy between measurements in whole pancreas and isolated islets is intriguing and cannot be resolved without further investigations. In summary, based on the present measurements and previous morphological studies^20^, we propose that the excessive secretion of glucagon in T2D results from a defective control of α-cell function rather than from changes in α-cell mass and glucagon stores.

In ND subjects, pancreatic somatostatin concentration averaged 0.46 µg/g, a value that is close to the 0.60 µg/g found in infants^48^, but 5-fold lower than in previous measurements in adult pancreas^14^. It did not correlate with age or BMI. Concentrations measured in T2D subjects overlapped those in ND subjects. Although the total content was 29% lower in T2D subjects, the ratio between somatostatin and insulin or glucagon concentrations was unchanged. No differences have been observed in the volume density or mass of δ-cells between T2D and ND subjects^23, 41, 49^ except in one study reporting a 27% decrease in T2D subjects^18^. Although these biochemical and morphological approaches do not suggest major alterations in δ-cell mass and somatostatin stores, further studies are needed to determine whether altered somatostatin secretion plays a significant (paracrine) role in the dysfunction of α- and β-cells in type-2 diabetes.

## Conclusion

Pancreatic insulin stores are reduced by 30–35% in T2D compared to ND subjects, whereas glucagon stores are unchanged. In general, these biochemical findings agree with reported changes of β- and α-cell mass in the pancreas. Together, they point to the importance of functional defects in the insufficient secretion of insulin and excessive secretion of glucagon in type-2 diabetes. Not all comparisons between hormone contents in the pancreas and isolated islets are in agreement, indicating that caution must be exercised before extrapolating data obtained in selected islets to the whole islet population. Ideal experiments should compare cell proportions and hormone concentrations in parallel samples of whole pancreas and isolated islets.

## Methods

### Subjects

The study was conducted according to the regulations of the Commission d’Ethique Biomédicale of the Faculty of Medicine of the University of Louvain in Brussels. Hormone concentrations were measured in the pancreas of 57 European subjects, who underwent an autopsy within 12 h of death at the University Hospital Saint-Luc in Brussels between 1985 and 1989. All extractions and assays were completed in 1989–1990, but the results were inexplicably consigned to oblivion until very recently. Subjects were assigned to the T2D group on the basis of their clinical history and use of an antidiabetic treatment for several months or years. The group of ND subjects was composed of individuals without a clinical history of diabetes. Unfortunately, for a number of these ND subjects, no definitive proof of the absence of diabetes (such as normal blood glucose or HBA1c levels) could be traced owing to the long delay between acquisition of the data and their recent analysis. Ten subjects of the present series of 57 were also included in the cases whose pancreatic insulin concentration was reported in a previous publication that did not analyze glucagon or somatostatin concentrations^17^.

The whole group of 39 ND subjects was composed of two subgroups of 20 lean subjects (LND, BMI < 25) and 19 obese subjects (OND, BMI ≥ 27). The group of LND subjects (15 M/5 F) had a mean age of 62.4 years (range 28–86) and a mean BMI of 22.5 (19.2–24.7). The group of OND subjects (13 M/6 F) had a mean age of 63.8 years (range 33–87) and a mean BMI of 32.4 (27.0–46.6). The group of T2D subjects (12 M/6 F) had a mean age of 66.2 years (range 54–84) and a mean BMI of 29.9 (19.5–48.5). Gender proportions and ages were not different between groups. The BMI of T2D subjects was higher than that of LND subjects (P < 0.001) but not different from that of OND subjects or the whole group of ND subjects. The time between diagnosis of T2D and death was known in 14/18 cases and averaged 10.5 y (0.5–22). Eight of the T2D subjects received insulin (combined with an oral drug in 4 cases) and 10 were on oral drug only (2 on metformin and 8 on sulfonylureas). Clinical characteristics of the studied subjects are given in Supplementary Table 1.

### Pancreas processing

Only pancreases without macroscopic or microscopic signs of autolysis were studied. After resection, the pancreas was trimmed of adherent fat and mesenchymal tissue and weighed. A fragment was then taken from the body, weighed and frozen until hormone extraction. These fragments of about 1 g were finely minced, homogenized in acid-ethanol and sonicated three times after 24-h periods of storage at −20 °C. Hormone concentrations in extracts were measured by radioimmunoassays using a) human mono-component insulin (Novo Biolabs, Bagsvaerd, Denmark) and insulin antiserum L619 that cross-reacted with proinsulin (obtained from P.H. Wright, Indianapolis, U.S.A.), (b) porcine glucagon (Novo) and pancreatic glucagon antiserum 30 K (obtained from R.H. Unger, Dallas, U.S.A.), and (c) synthetic somatostatin-14 and an antiserum against it (U.C.B., Braine-L’Alleud, Belgium).

### Presentation of results

Results are presented as scatter plots of individual values or as means ± SD. The number of measurements sometimes slightly differed from the total number of subjects because somatostatin was not measured in 1/20 LND, 3/19 OND and 4/18 T2D cases. The statistical significance of differences between groups was assessed by Student’s t test when only two groups were compared and by Anova followed by a Newman-Keuls test for multiple comparisons. Results were also analyzed using non-parametric tests: Mann-Whitney test to compare two groups and Kruskal-Wallis test followed by a Dunn test for multiple comparisons. The few discrepancies between parametric and non-parametric analyses are mentioned in Figure legends. Correlations between pancreas weight or hormone content and age or BMI were assessed by the test of Spearman.

### Data availability

All data generated or analyzed during this study are included in this published article and its Supplementary Table and Figure.

## Electronic supplementary material

Supplementary Table 1 and Figure 1
