# Supplementary material for: Insulin, glucagon and somatostatin stores in the pancreas of subjects with type-2 diabetes and their lean and obese non-diabetic controls
Source: Sci Rep. 2017 Sep 8;7:11015. doi: 10.1038/s41598-017-10296-z (PMC5591190; doi:10.1038/s41598-017-10296-z)
Supplement: Supplementary file 1 — Supplementary Table 1 and Figure 1 [file 41598_2017_10296_MOESM1_ESM.pdf]

Insulin, glucagon and somatostatin stores in the pancreas of subjects with type-2 diabetes and their lean and obese non-diabetic controls

Jean-Claude Henquin, Majeed M. Ibrahim and Jacques Rahier

Henquin JC et al: Supplementary Table 1

LND : Lean Non-Diabetic subjects

	Sex	Age (y)	BMI	Diabetes Years	Diabetes Treatment	Cause of death
LND 1	M	28	22.5			Wilson disease
LND 2	M	35	23.0			Acute myeloid leukemia
LND 3	M	40	20.3			Traffic accident
LND 4	M	40	21.9			Renal insufficiency
LND 5	M	50	23.9			?
LND 6	F	52	23.6			?
LND 7	M	58	24.4			Traffic accident
LND 8	M	59	23.3			?
LND 9	M	61	23.2			Mitral and aortic valves
LND 10	M	65	24.3			Post-surgery cardiac arrest
LND 11	F	72	19.5			Pneumonia and septicemia
LND 12	M	73	24.0			Myocardial Infarction
LND 13	M	73	24.7			Myocardial Infarction
LND 14	M	74	21.0			Acute renal insufficiency
LND 15	M	74	21.5			Gastrointestinal haemorrhage
LND 16	F	77	23.6			Myocardial infarction
LND 17	M	76	19.2			?
LND 18	M	77	22.2			Lung carcinoma
LND 19	F	78	20.4			Larynx carcinoma
LND 20	F	86	23.0			Subphrenic abcess

OND : Obese Non-Diabetic subjects

	Sex	Age (y)	BMI	Diabetes Years	Diabetes Treatment	Cause of death
OND 1	M	33	28.4			Methanol intoxication
OND 2	M	43	30.0			Cardiomyopathy
OND 3	M	44	27.2			Congestive cardiomyopathy
OND 4	F	52	33.4			Breast cancer
OND 5	F	60	46.6			?
OND 6	F	61	28.5			Bronchopneumonia
OND 7	F	62	32.6			Myocardial infarction
OND 8	M	63	31.0			Acute myeloid leukemia
OND 9	M	63	39.4			Post-surgery cardiac arrest
OND 10	F	64	39.3			Gastrointestinal haemorrhage
OND 11	M	66	27.7			Mitral valve insufficiency
OND 12	M	67	27.5			?
OND 13	M	69	31.3			Myocardial infarction
OND 14	M	74	27.0			Aortic valve stenosis
OND 15	M	74	29.4			Acute respiratory distress
OND 16	M	76	32.5			?
OND 17	F	76	45.3			?
OND 18	M	78	28.5			Aortic aneurysm
OND 19	M	87	29.1			Stroke

T2D : Type 2 Diabetic subjects

	Sex	Age (y)	BMI	Diabetes Years	Diabetes Treatment	Cause of death
T2D 1	M	50	32.5	1	Metformin	Congestive cardiomyopathy
T2D 2	M	54	26.0	?	Insulin	Cardiogenic shock
T2D 3	M	54	32.0	13	SU* + Insulin	Werner syndrome - Pneumonia
T2D 4	M	55	35.6	5	Insulin	Lymphoma - Septic shock
T2D 5	F	56	35.5	7	SU	Post-surgery complications
T2D 6	M	57	33.6	2	SU	Intestinal infarction
T2D 7	F	58	26.4	10	SU	Myocardial infarction
T2D 8	M	64	25.7	22	Insulin	Myocardial Infarction
T2D 9	F	65	25.7	17	SU	Myocardial Infarction
T2D 10	M	65	30.0	?	SU + Insulin	Cerebral hemorraghe
T2D 11	M	71	30.7	10	SU	Acute Respiratory Distress
T2D 12	F	72	48.5	13	SU + Insulin	Asphyxia - Morphine
T2D 13	M	75	20.2	5	SU	Pneumonia
T2D 14	F	75	25.0	?	SU	?
T2D 15	M	75	25.7	21	SU + Insulin	Sudden cardio-resp arrest
T2D 16	M	80	28.7	0.5	Metformin	Bronchopneumonia
T2D 17	M	81	25.7	21	SU	Perfor peptic ulcer + Shock
T2D 18	F	84	19.5	?	Insulin	Myocardial Infarction

* SU = Sulfonylurea

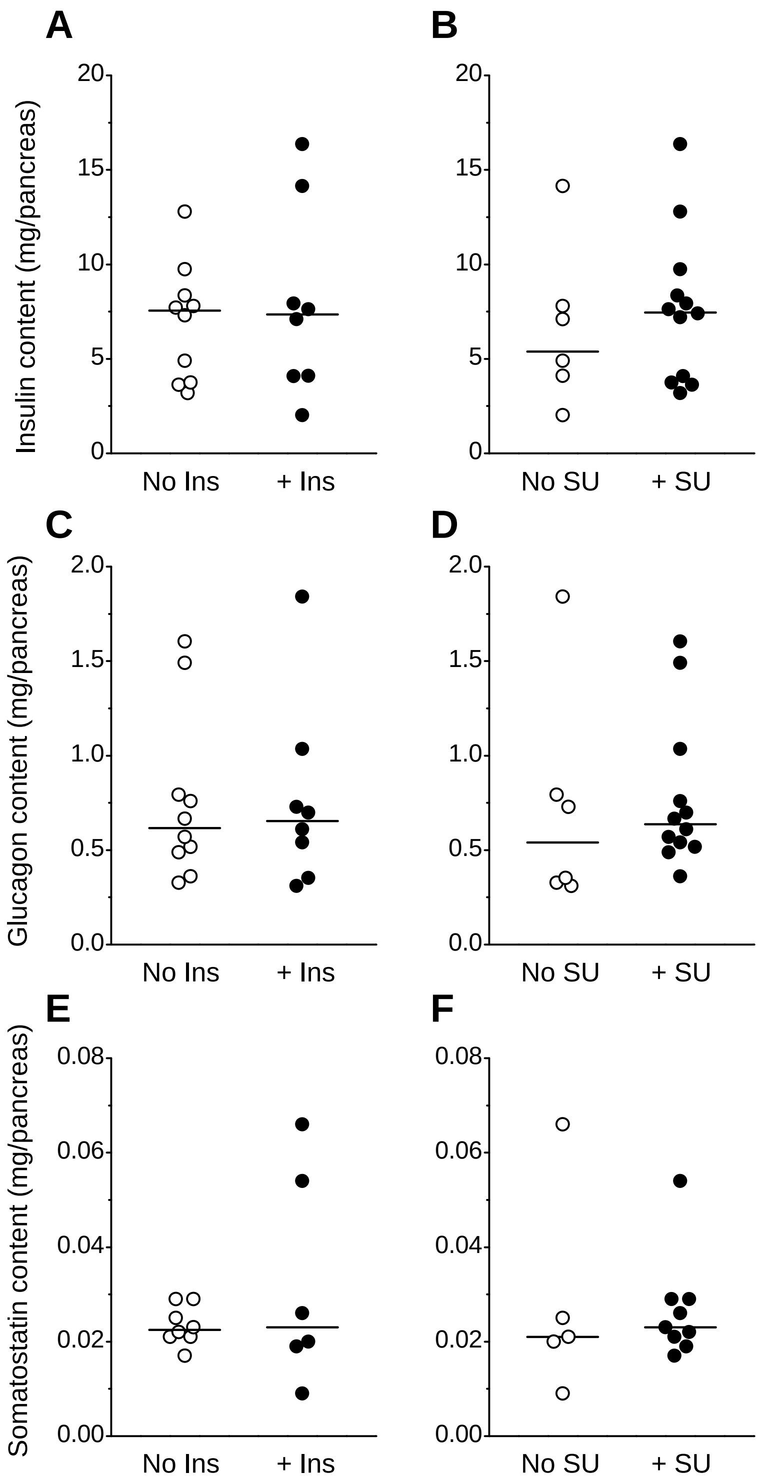

Supplementary Figure 1: Impact of treatment type on insulin (A-B), glucagon (C-D) and somatostatin (E-F) content in the pancreas of type 2 diabetic subjects. A,C,E: The 18 subjects were subdivided in two groups whose treatment included insulin injections (+ Ins) or not (No Ins). B,D,F: The subjects were subdivided in two groups whose treatment included a sulfonylurea (+ SU) or not (No SU). Details are given in Supplementary Table 1. Individual values are shown with median. There was no significant difference between groups.
